# Transcriptome Profile of Near-Isogenic Soybean Lines for β-Conglycinin α-Subunit Deficiency during Seed Maturation

**DOI:** 10.1371/journal.pone.0159723

**Published:** 2016-08-17

**Authors:** Bo Song, Lixin An, Yanjing Han, Hongxiu Gao, Hongbo Ren, Xue Zhao, Xiaoshuang Wei, Hari B. Krishnan, Shanshan Liu

**Affiliations:** 1 Key Laboratory of Soybean Biology in Chinese Ministry of Education (Key Laboratory of Soybean Biology and Breeding/Genetics of Chinese Agriculture Ministry), Northeast Agricultural University, Harbin 150030, China; 2 Inspection and Testing Center for Quality of Cereals and their Products, Ministry of Agriculture China, Harbin 150030, China; 3 Plant Genetics Research Unit, Agricultural Research Service (ARS), United States Department of Agriculture, University of Missouri, Columbia, Missouri, 65211, United States of America; 4 Department of allergy, First Affiliated Hospital, Harbin Medical University, Harbin, China; National Institute for Plant Genome Research, INDIA

## Abstract

Crossing, backcrossing, and molecular marker-assisted background selection produced a soybean (*Glycine max*) near-isogenic line (*cgy-2-*NIL) containing the *cgy-2* allele, which is responsible for the absence of the allergenic α-subunit of β-conglycinin. To identify α-null-related transcriptional changes, the gene expressions of *cgy-2-*NIL and its recurrent parent DN47 were compared using Illumina high-throughput RNA-sequencing of samples at 25, 35, 50, and 55 days after flowering (DAF). Seeds at 18 DAF served as the control. Comparison of the transcript profiles identified 3,543 differentially expressed genes (DEGs) between the two genotypes, with 2,193 genes downregulated and 1,350 genes upregulated. The largest numbers of DEGs were identified at 55 DAF. The DEGs identified at 25 DAF represented a unique pattern of GO category distributions. KEGG pathway analyses identified 541 altered metabolic pathways in *cgy-2-*NIL. At 18DAF, 12 DEGs were involved in arginine and proline metabolism. The *cgy-2* allele in the homozygous form modified the expression of several Cupin allergen genes. The *cgy-2* allele is an alteration of a functional allele that is closely related to soybean protein amino acid quality, and is useful for hypoallergenic soybean breeding programs that aim to improve seed protein quality.

## Introduction

Soy-seed-derived products and their nutritional quality are affected by the subunit composition of seed storage proteins [1–4]. Glycinin (11S globulin) and β-conglycinin (7S) are the two main proteins in soybean seeds, accounting for ~70% of total seed proteins. By manipulating the identified variant alleles of glycinin and β-conglycinin, it is possible to breed soybean varieties with modified protein compositions, ranging from extremely high to extremely low 11S:7S ratios, which have led to improved nutritional values and food-processing properties [1,5–6]. In the past three decades, efforts to develop 7S-low-type soybean lines have led to the availability of various 7S or 11S globulin protein subunit null varieties among soybean germplasms [1,7–12]. Despite the deficiency of 7S and 11S major protein subunits, the nitrogen content of the mutant dry seeds is similar to (or higher than) wild-type cultivars, and most mutants grow and reproduce normally [2]. Specifically, β-conglycinin allergen-subunit-deficiency mutants have high nutritional value and low allergenic risk [1,5–6,13–14].

β-Conglycinin is the major seed protein of soybean (*Glycine max* (L.) Merr.), and comprises three subunits: α′ (76 kDa), α (72 kDa), and β (52 kDa) in varying proportion [15]. β-Conglycinin contains lower amounts of sulfur-containing amino acids and has a reduced gel-forming ability than glycinin [16]. Specifically, the α and α′-subunits of β-conglycinin negatively influence the nutrition of seed proteins and the gelation of tofu [1,5,12,17]. In addition, the three subunits are major allergens [5,17–20]. Genetic studies demonstrated that the absence of the α-subunit is controlled by a single recessive α-null allele, *cgy-2* [21–23]. Gene symbols *Cgy2*/*cgy2* were proposed for the genes that confer the presence or absence of the α-subunit of soybean β-conglycinin [21–22]. To date, the genetic effect of the α-null mutation and the molecular mechanism of *cgy-2* allele variation remain unclear.

The transcriptome corresponding to most of the protein coding genes is a small but important representation of the genome. Recently, RNA sequencing (RNA-seq) technologies have been developed that offer an opportunity to deliver fast, cost-effective, and accurate means to analyze the transcriptome in non-model organisms. With advances in RNA-seq, a large number of molecular markers and transcripts involved in specific biological processes could be identified. In soybean, transcriptome analyses of gene expression profiles during soybean seed development have been conducted mainly using microarray analysis and RNA-seq technology [24–27]. By utilizing DNA microarray analysis, Narikawa et al. [24] verified the changes in seed metabolism in the glycinin-null cultivar Tousan 205. Tousan 205 exhibited higher expression levels of stress-related genes, such as ascorbate peroxidase, than its parent cultivar ‘Tamahomare’. Their results suggested that the deficiency of glycinin caused an expression change of stress-related genes.

In contrast to the *Cgy-2* allele (conferring α-normal), information on the *cgy-2* allele (conferring α-null) is limited. In the present study, we have examined the effect of *cgy-2* allele on the amino acid composition and gene expression. The information generated from this study will be valuable to soybean breeders involved in the modification of soybean seed protein composition.

## Materials and Methods

### Plant materials

**Fig 1:**
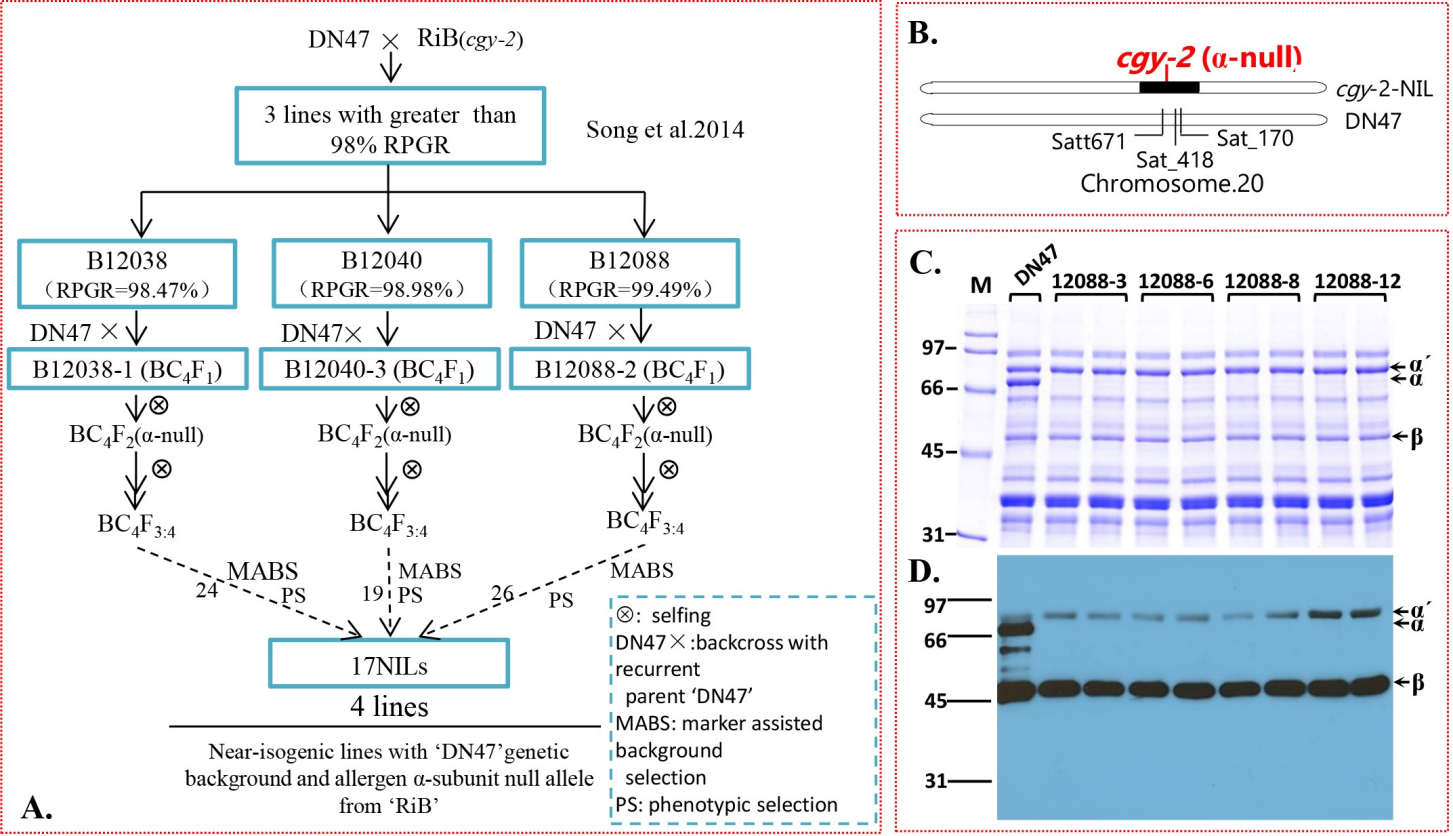
(A) Breeding process of the ‘*cgy-2-*NIL’. RPGR = recurrent parent genome recovery. Discontinuous lines link the last generation where the definitive near isogenic lines (NILs) were selected. Numbers alongside discontinuous lines show how many lines were obtained in each candidate line. (B) Graphical genotype for chromosome 20 in ‘*cgy-2-*NIL’ with the ‘DN47’ genetic background. (C) SDS-PAGE analysis of mature seed proteins of DN47 and four *cgy-2-*NIL lines: B-12088-3, B-12088-6, B-12088-8, and B-12088-12. (D) Immunoblot analysis of the seed extracts shown in (C) using antibodies specific for β-conglycinin subunits. The sizes of the protein markers (M) in kilodaltons are shown on the left of the image in (C). The arrows point to the α′, α, and β-subunits of β-conglycinin in Fig (C) and (D).

Near-isogenic line (NIL) *cgy-2-*NIL, carrying the *cyg-2* allele (conferring α-null) (Fig 1), used for this study were derived from an α-subunit-null population, which has previously been used by our group to develop α-subunit-null improved lines with a Chinese soybean genetic background [12].

**Fig 2:**
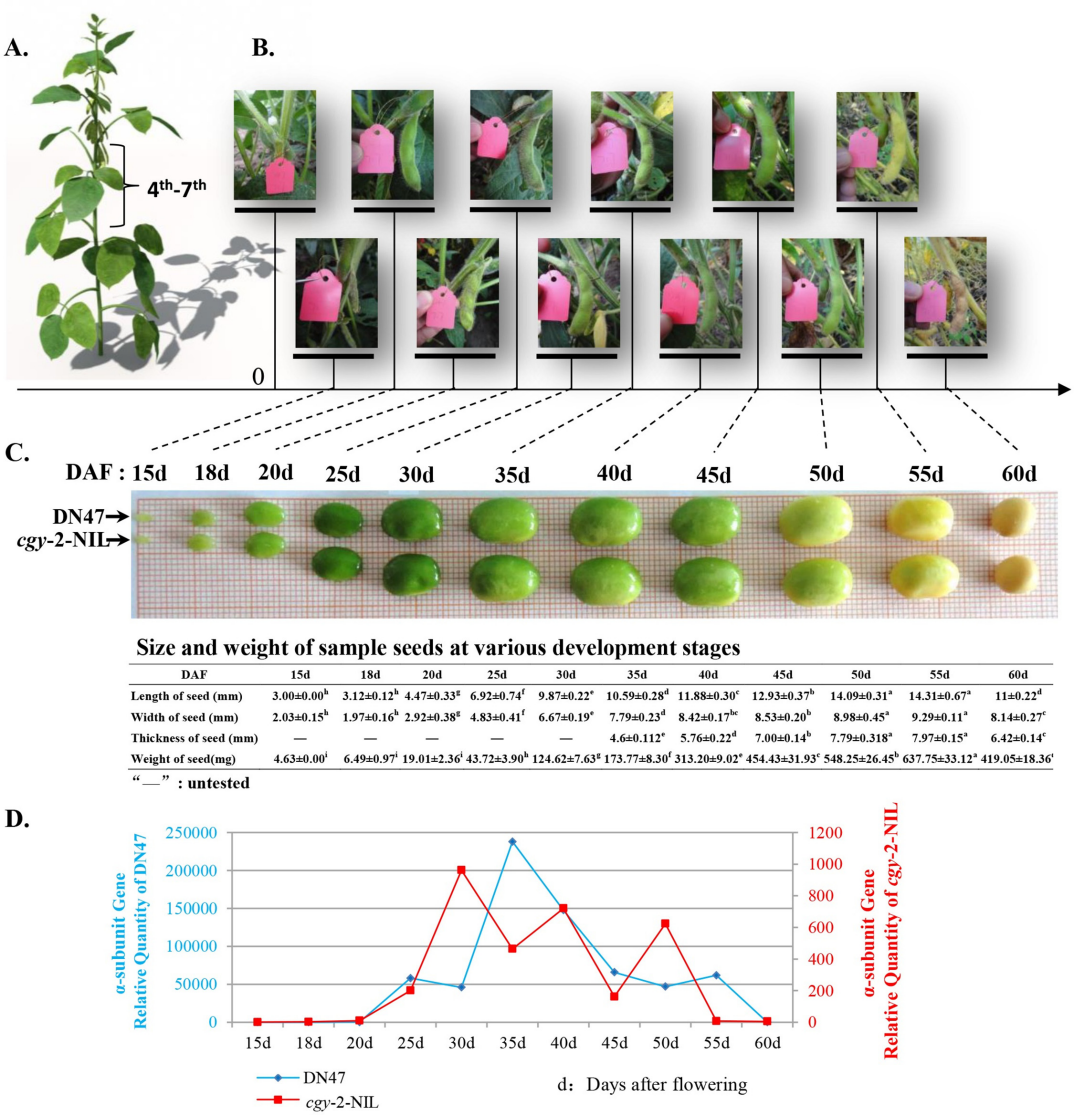
(A) Fully expanded flowers were marked individually with a tag at the 4^th^, 5^th^, 6^th^, or 7^th^ nodes on *cgy-2*-NIL and DN47. (B) Pod samples were collected during seed development at 15, 18, 20, 25, 30, 35, 40, 45, 50, 55, and 60 days after flowering (DAF). (C) Developmental changes in morphology, size, and weight of sampled seeds in *cgy-2*-NIL and DN47. (D) Comparison of transcript levels of the α-subunit gene between *cgy-2*-NIL and DN47. Differential expression of more than 50-fold was identified during the 25–55 days after flowering (DAF) development stages. Five stages of soybean seeds collected at 18, 25, 35, 50, and 55 DAF were finally selected to subjected to RNA-seq.

About 45 days after sowing, fully expanded flowers were marked individually with a tag at the 4^th^, 5^th^, 6^th^, or 7^th^ nodes on *cgy-2*-NIL and DN47 (Fig 2A). Pod samples were collected during seed development at 15, 18, 20, 25, 30, 35, 40, 45, 50, 55, and 60 days after flowering (DAF, Fig 2B) during the summer of 2014. All seed samples (BC_4_F_5_) (combined cotyledon and seed coat) of a given age were pooled and stored at −80°C for future use (Fig 2C). Unusual-sized seeds were excluded from the soybean samples. Based on the assessment of the different expressions of the α-subunit gene between the *cgy-2-*NIL and DN47 by quantitative real-time reverse transcription PCR (qRT-PCR) (Fig 2D), five stages of soybean seeds collected at 18, 25, 35, 50, and 55 DAF were finally selected for RNA-seq analysis.

### SDS-PAGE and immunoblot analysis

Sodium dodecyl sulfate polyacrylamide gel electrophoresis (SDS-PAGE) and immunoblot analysis were performed as described earlier [20, 28]. Briefly, total seed proteins (25 μg) from DN47 and *cgy-2*-NIL lines were resolved on 10% polyacrylamide gels. Separated proteins were visualized by staining with Coomassie brilliant blue or electrophoretically transferred to a nitrocellulose membrane and incubated with polyclonal antibodies raised against the α-subunit of β-conglycinin. Immunoreactive proteins were detected using an anti-rabbit IgG-horseradish peroxidase conjugate followed by chemiluminescent detection.

### Determination of seed protein content and amino acid analysis of ‘*cgy-2*-NILs’

Dry seeds of DN47 and *cgy-2*-NIL were harvested at maturity in 2014 and stored at room temperature. Ten plants of each *cgy-2-*NIL and DN47 were examined. Total seed nitrogen was measured using the Micro-Kjeldahl method (Foss, 2300 Kjeltec Analyzer Unit). The crude protein content was determined by calculating the nitrogen content and then multiplying the result by a conversion factor of 6.25.

Total amino acids (AAs) were obtained from hydrolysis of seed meal in 6 M HCl for 22 h in sealed evacuated tubes at a constant boiling temperature of 110°C. An amino acid analyzer (Hitachi L-8800; Hitachi, Tokyo, Japan) was used to determine the AA composition of the hydrolysates.

Free AAs were extracted from 5.00 g of seed meal. Seed meal (seeds were sampled using a sample quartiles method, fully dried with mill grinding through a 0.25-mm sieve, and thoroughly mixed) was finely homogenized in 30 mL of sulfosalicylic acid (10 g per 100 mL) and disrupted ultrasonically for 30 min. The supernatant was centrifuged at 5000 × *g* for 5 min. The resultant supernatant was filtered through a 22-μm GD/X sterile disposable syringe filter. A Hitachi L-8800 amino acid analyzer was then used to analyze the filtrate.

The amino acid quality was compared between *cgy-2-*NIL and DN47 using a scoring method. The amino acid score (AAS) was calculated according to the scoring pattern suggested by the Food and Agriculture Organization and World Health Organization (FAO/WHO) [29]. Concentration was expressed as grams of amino acid/16 gN in the test protein divided by grams of amino acid/16 gN in the scoring pattern. Each data set and reference patterns were also used to calculate EAAI (essential amino acid index) [30, 31]. The EAAI is the geometric mean of the individual amino acid scores and is equal to the antilogarithm of the individual scores. The AAS was calculated using the following formula:

$$\mathrm{Amino acid score}=\frac{\mathrm{mg of amino acid in}\;1\;\mathrm{g of test protein}}{\mathrm{mg of amino acid in}\;1\;\mathrm{g reference pattern}}\times 100$$

The EAAI values were assigned a maximum of 1.00 and a minimum of 0.01. Feedstuffs are rated as good-quality protein sources when the EAAI is ≥ 0.90, adequate when approximately 0.80, and inadequate below 0.70 [32].

### RNA isolation, cDNA library construction, and Illumina deep sequencing

Seed samples harvested at five growth stages corresponding to 18, 25, 35, 50, and 55 DAF from DN47 and *cgy-2*-NIL in the summer of 2014 were used for RNA-seq analysis. Two individual biological replicates were tested for the five developmental stages, resulting in 20 samples. In order to minimize biological variation, RNA from separate biological samples was used for the two biological replicates per stage, the values of correlation coefficient (R^2^ value) of all DEGs for each 2 biological replicates ranged from 0.961 to 0.992. All of the samples were stored in liquid nitrogen immediately after collection in the field and then transported to a −80°C freezer in our laboratory at the Northeast Agriculture University soybean research center.

Total RNA was extracted from each sample using the improved cetyl trimethylammonium bromide method [33]. RNA degradation and contamination was monitored on 1% agarose gels. A NanoPhotometer spectrophotometer (Implen, CA, USA) was used to check the RNA purity. A Qubit RNA Assay Kit in a Qubit 2.0 Fluorometer (Life Technologies, CA, USA) was used to measure the RNA concentration and an RNA Nano 6000 Assay Kit of the Bioanalyzer 2100 system (Agilent Technologies, CA, USA) was utilized to assess the RNA integrity. All RNA samples had RNA integrity number (RIN) values above 6.5.

mRNA was extracted using Dynabeads oligo (dT) (Dynal; Invitrogen). Double-stranded cDNAs were synthesized using reverse transcriptase (Superscript II; Invitrogen) and random hexamer primers. To select preferentially cDNA fragments of 200 bp in length, the library fragments were purified using the AMPure XP system (Beckman Coulter, Beverly, CA, USA). DNA fragments with ligated adaptor molecules on both ends were enriched selectively using the Illumina PCR Primer Cocktail in a 10-cycle PCR reaction. Products were purified using the AMPure XP system and quantified using the Agilent high-sensitivity DNA assay on the Agilent Bioanalyzer 2100 system. cDNA Library concentration was first quantified using a Qubit 2.0 fluorometer (Life Technologies), and then diluted to 1 ng/μl before checking insert size on an Agilent 2100 and quantifying to greater accuracy by quantitative PCR (Q-PCR) (library activity >2 nM).The library preparations were sequenced on an Illumina Hiseq 2000 platform and 100-bp paired-end reads were generated. Illumina sequencing was performed by Novogene Bioinformatics Technology Co., Ltd., Beijing, China (www.novogene.cn).

### Bioinformatic analysis of differentially expressed genes (DEGs)

To obtain high-quality clean reads, raw data (raw reads) in fastq format were first processed using in-house Perl scripts. The calculation of Q20, Q30, GC-content, and all the downstream analyses were based on the high-quality clean data. The reference genome (ftp://ftp.ensemblgenomes.org/pub/release-23/plants/fasta/glycine_max/) and gene model annotation files were downloaded from the genome website directly. We used HTSeq v 0.6.1 (www-huber.embl.de/users/anders/HTSeq/) to count the read numbers mapped to each gene. Data were then provided in reads per kilobase per million reads (RPKMs) [34]. Differential expression analysis was performed using the DESeqR package (1.10.1) [35]. P-values were adjusted using the Benjamini and Hochberg approach; with a P-value < 0.05 being used as the threshold for significant differential expression. Gene Ontology (GO) (http://www.geneontology.org/) analysis was performed by the GOseq R package [36], and GO terms with corrected P-values < 0.05 were considered significantly enriched for the DEGs. We used KOBAS software (KOBAS, Surrey, UK) to test the statistical enrichment of DEGs in KEGG pathways (http://www.kegg.jp/kegg/pathway.html). Datasets were deposited in the GEO (http://www.ncbi.nlm.nih.gov/geo/query/acc.cgi?token=qpwjwcsqjhahzed&acc=GSE79327) with accession number GSE79327.

### qRT-PCR confirmation of the Illumina sequencing data

**Fig 3:**
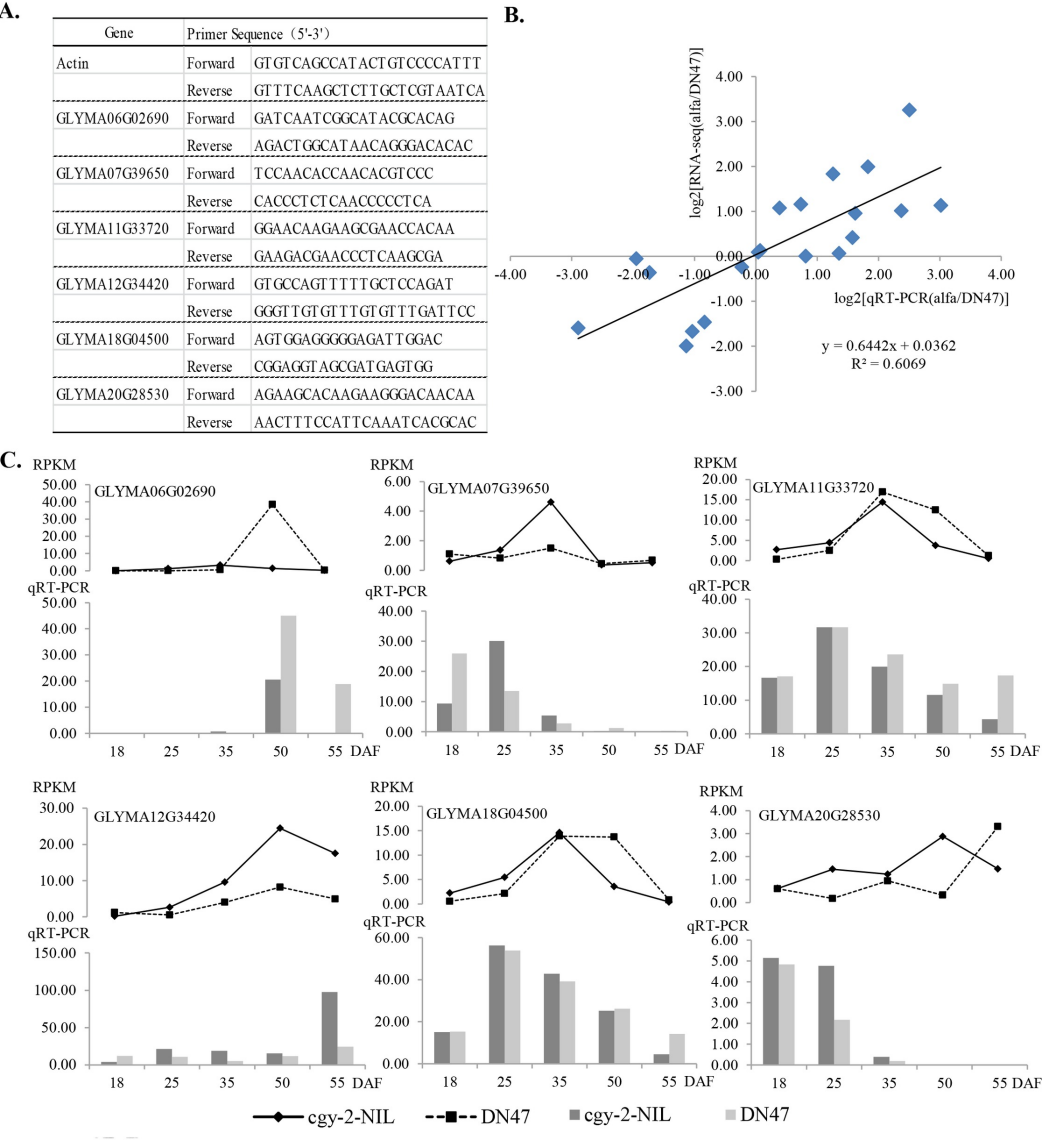
(A) Primer sequences used in quantitative real-time reverse transcription PCR (qRT-PCR) analysis for validation of the expressed genes in Illumina sequencing. (B) Comparison between the gene expression ratios obtained from RNA-seq data and that from qRT-PCR. The RNA-seq log2 value of the expression ratio (y-axis) was plotted against the developmental stages (x-axis). (C) qRT-PCR analysis of differentially expressed genes between *cgy-2*-NIL and DN47. The transcript abundance from the RNA-seq data is shown at the top of the panel for each gene. RPKM: reads per kilobase per million reads. DAF = days after flowering.

RNA-seq data were further validated using qRT-PCR for six selected genes, using gene-specific primer sets (Fig 3A). Primer pairs were designed using the Primer 5 software. Actin was amplified along with the target gene as an endogenous control to normalize expression between different samples. qRT-PCR was performed using a real-time RT-PCR kit (Takara, Japan), on a CFX96 Real-Time System (BioRad, USA). The delta-delta-cycle threshold (Ct) method was used to calculate the relative expression of each mRNA [37].

## Results

### Phenotype screening for α-subunit nulls using SDS-PAGE and immunoblot analysis

The *cgy-2-*NILs were derived as outlined in Fig 1A. In the BC_3_F_2_ population [12], three individuals, B12038, B12040, and B12088, with recurrent parent genome recoveries of 98.47%, 98.98%, and 99.49%, respectively, were selected as α-null donor parents. BC_4_F_2_ progeny derived from these selected BC_3_F_2_ parents with the homozygous *cgy-2* gene, were selfed to obtain 69 BC_4_F_3:4_ individuals, which were designated as *pre-ILs*, including three sets: 24 lines for B12038, 19 lines for B12040, and 26 lines for B12088 (Fig 1A). These *pre-ILs* were genotyped again using polymorphic molecular markers for verification. Uniformity regarding plant type was also examined within each line. Seventeen lines containing only a single introgression in chromosome 20 containing the *cgy-2* gene in B12088 progeny were obtained (Fig 1B). Combined with stringent phenotypic selection, a final collection of four ideal NILs was obtained, with each NIL containing the *cgy-2* allele (hereafter named ‘*cgy-2-*NIL’) (Fig 1A). BC_4_F_5_ seeds obtained in 2014 from the above experiments were used for all subsequent experiments in this study. The recurrent parent DongNong 47 (DN47), containing all storage protein subunits, was used as the control (Fig 1C, lane 1). *cgy-2-*NIL only differed from the DN47 control by its introgressed donor DNA fragment containing the *cgy-2* gene (Fig 1B). Therefore, any observable phenotypic differences are expected to be a result of the *cgy-2* gene.

The absence α-subunit of β-conglycinin in the developed *cgy-2-*NIL was verified by SDS-PAGE (Fig 1C) and immunoblot analysis (Fig 1D). An examination of the total seed protein profile of DN47 revealed the presence of all three subunits of β-conglycinin (Fig 1C). In contrast, the *cgy-2*-NILs (12088–3, 12088–6, 12088–8, and 12088–12) failed to accumulate the 72-kDa α subunit of β-conglycinin (Fig 1C). This observation was further confirmed by western blot analysis (Fig 1D). β-conglycinin-specific antibodies recognized all the three subunits of β-conglycinin from DN47, while the *cgy-2*-NILs showed no reactivity against the 72-kDa α subunit of β-conglycinin. This observation confirms that the α subunit of β-conglycinin is absent in the *cgy-2*-NILs.

### Effect of allelic variation of the α-subunit locus on soybean amino acid composition

To understand the effect of allergen-α-subunit-deficiency on soybean amino acids (AAs) composition, the AA content and nutritional quality were investigated. The crude protein content, AA concentration, and free amino acids (FAA) concentrations of the homozygous *cgy-2*-NIL and the recurrent parent DN47 were compared. In *cgy-2-*NIL compared with DN47, there was a 4.11%, 4.16%, 5.20%, and 11.96% increase in crude protein content, total AA content, total essential amino acid (TEAA) content, and sulfur-containing (Met and Cys) content, respectively (Table 1). The concentration of Thr, Val, Met, and Ile increased significantly in *cgy-2-*NIL, resulting in a significant increase in TEAA content. The sulfur-containing (Met and Cys) AA concentration increased significantly in *cgy-2-*NIL. The total AA concentration also increased in *cgy-2-*NIL because of the general increase in constituent content of most AAs (Table 1).

**Table 1:** Comparison of amino acid and free amino acid contents of mature seeds between ‘DN47’ and its near-isogenic line,‘*cgy-2* NIL’.

	A.A.		F.A.A.	
	D47 (%)	NIL (%)	D47 (mg/g)	NIL (mg/g)
Essential amino acids				
Thr	1.38 ± 0.09	1.54 ± 0.03*	0.2127 ± 0.0115	0.2783 ± 0.0084*
Val	1.48 ± 0.04	1.55 ± 0.02*	0.1007 ± 0.0167	0.1043 ± 0.0032
Met	0.40 ± 0.06	0.52 ± 0.01*	0.0660 ± 0.0035	0.0840 ± 0.0010*
Ile	1.48 ± 0.06	1.58 ± 0.02*	0.0720 ± 0.0069	0.0707 ± 0.0029
Leu	2.71 ± 0.05	2.80 ± 0.04	0.1133 ± 0.0098	0.1510 ± 0.0046*
Phe	1.80 ± 0.04	1.79 ± 0.03	0.1480 ± 0.0017	0.1677 ± 0.0025*
Lys	2.29 ± 0.03	2.36 ± 0.04	0.2287 ± 0.0271	0.2537 ± 0.0012
T.E.A.A.	11.53 ± 0.24	12.13 ± 0.16*	0.9413 ± 0.0577	1.1097 ± 0.0015*
Non-essential amino acids				
Asp	3.85 ± 0.12	4.06 ± 0.04*	0.7033 ± 0.0046	0.6263 ± 0.0381*
Ser	1.81 ± 0.06	1.90 ± 0.02	0.0825 ± 0.0009	0.1133 ± 0.0068*
Glu	5.93 ± 0.13	5.87 ± 0.04	0.5113 ± 0.0144	0.5020 ± 0.0156
Gly	1.45 ± 0.02	1.53 ± 0.03*	0.0633 ± 0.0058	0.0913 ± 0.0006*
Ala	1.47 ± 0.03	1.58 ± 0.02*	0.0940 ± 0.0052	0.1663 ± 0.0045*
Cys	0.52 ± 0.01	0.51 ± 0.01	0.1873 ± 0.0023	0.1797 ± 0.0075
Tyr	1.14 ± 0.03	1.19 ± 0.03	0.0873 ± 0.0023	0.1220 ± 0.0070*
His	0.89 ± 0.02	0.99 ± 0.01*	0.1367 ± 0.0289	0.2873 ± 0.0452*
Arg	2.36 ± 0.08	2.58 ± 0.02*	0.7547 ± 0.0214	1.9817 ± 0.3537*
Pro	1.77 ± 0.07	1.75 ± 0.02	0.1450 ± 0.0364	0.2043 ± 0.0015*
T.A.A	32.72 ± 0.41	34.08 ± 0.37*	3.7128 ± 0.0673	5.3837 ± 0.4481*
T.S.A.A.	0.92 ± 0.05	1.03 ± 0.01*	0.2533 ± 0.0058	0.2637 ± 0.0065
Protein	37.96 ± 0.39	39.52 ± 0.28*		

Data are means ± SD for seeds from at least three plants. Asterisks indicate statistically significant differences (*P < 0.05) between ‘DN47’ and ‘NIL-DN47-Δα’. Each amino acid is expressed using its three-letter abbreviation.

A.A.: amino acid; FAA: free amino acid; NIL: near-isogenic line’; T.A.A.: total amino acids; T.S.A.A: total sulfur-containing amino acids (Met+Cys).

The increased content of FAAs in *cgy-2-*NIL was most pronounced for Arg, which increased by more than two-fold compared with DN47 (Table 1). In *cgy-2-*NIL, Arg comprised 36.81% of FAAs, with Asp and Glu providing a further 11.63% and 9.32%, respectively; the remaining 42.24% comprised various other FAAs. His concentration also increased by two-fold in *cgy-2-*NIL; however, its content was much lower than Arg. The general and significant increase in the constituent content of most FAAs resulted in a significant increase in the total essential FAA and total FAA contents (Table 1).

The amino acid score was calculated according to the scoring pattern suggested by the FAO/WHO [29]. Both the total EAA content and the EAAI of *cgy-2-*NIL were higher than that of DN47 (Table 2). Our results suggested that the null allele of α-subunit positively affected the AA scores.

**Table 2:** Amino acid (A.A.) profile of mature seeds in ‘DN47’ and its near-isogenic line, ‘*cgy-2* NIL’.

A.A.	FAOmg/gPro.	D47		NIL (α-null)	
		mg/gPro.	A.A. Sco. (%)	mg/gPro.	A.A.Sco. (%)
Essential amino acids					
Thr	40	36.27	90.67	38.88	97.21
Val	50	38.90	77.80	39.30	78.61
Met+Cys	35	24.15	68.99	25.98	74.22
Ile	40	38.90	97.25	39.98	99.95
Leu	70	71.39	101.99	70.85	101.21
Phe+Tyr	60	77.45	129.08	75.40	125.67
Lys	55	60.33	109.68	59.63	108.42
Trp	10	-Not determined-			
TEAA	360	347.38		350.03	
EAAI(%)	100	79.25		82.16	
Non-essential amino acids					
Asp		101.42		102.73	
Ser		47.68		48.08	
Glu		156.13		148.45	
Gly		38.29		38.63	
Ala		38.64		39.90	
His		23.53		25.13	
Arg		62.08		65.20	
Pro		46.72		44.28	
TAA		861.87		862.43	

Data are expressed as means of triplicate experiments. Each amino acid is expressed using the three-letter code. A.A.: amino acid; Pro.: protein; A.A. Sco.: amino acid score; TEAA: total essential amino acids; EAAI: essential amino acid index; NIL: near-isogenic line *cgy*-2-NIL; TAA: total amino acids.

### DEGs between ‘*cgy-2-*NIL’ and ‘DN47’

**Fig 4:**
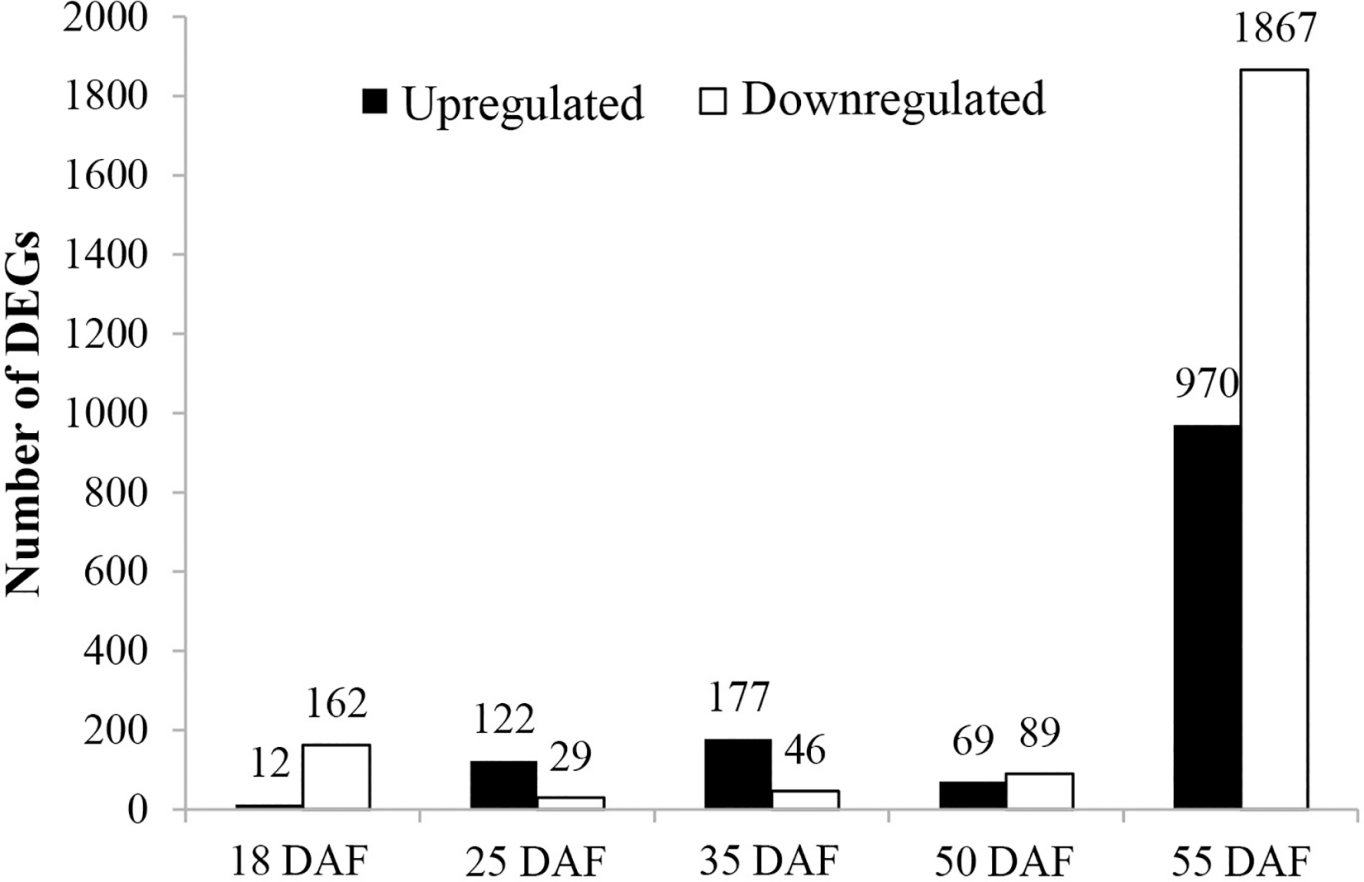
The number of differentially expressed genes (DEGs) between ‘*cgy-2*-NIL’ and ‘DN47’ at various developmental stages (18, 25, 35, 50, and 55 days after flowering (DAF)). Numbers of up and downregulated genes are summarized.

One of the primary goals of transcriptome sequencing is to compare the gene expression levels in two genotypes. A P-value < 0.05 and log2 (fold change) > 2 were used as the thresholds to judge the significant differences (enriched or depleted) in the gene expression profiles between *cgy-2-*NIL and DN47 at the same stage. Using these criteria, 20,295 DEGs were identified, which could be subdivided into 174, 151, 123, 158, and 2837 genes that varied in abundance at 18, 25, 35, 50, and 55 DAF, respectively (Fig 4). In general, throughout the five seed development stages, the total number of upregulated genes was less than the number of downregulated genes (Fig 4). Surprisingly, the maximum number of DEGs between *cgy-2-*NIL and DN47 was observed at 55 DAF. Furthermore, different from the other three stages (18, 50, and 55 DAF), there were more upregulated DEGs than downregulated DEGs at 25 and 35 DAF.

**Fig 5:**
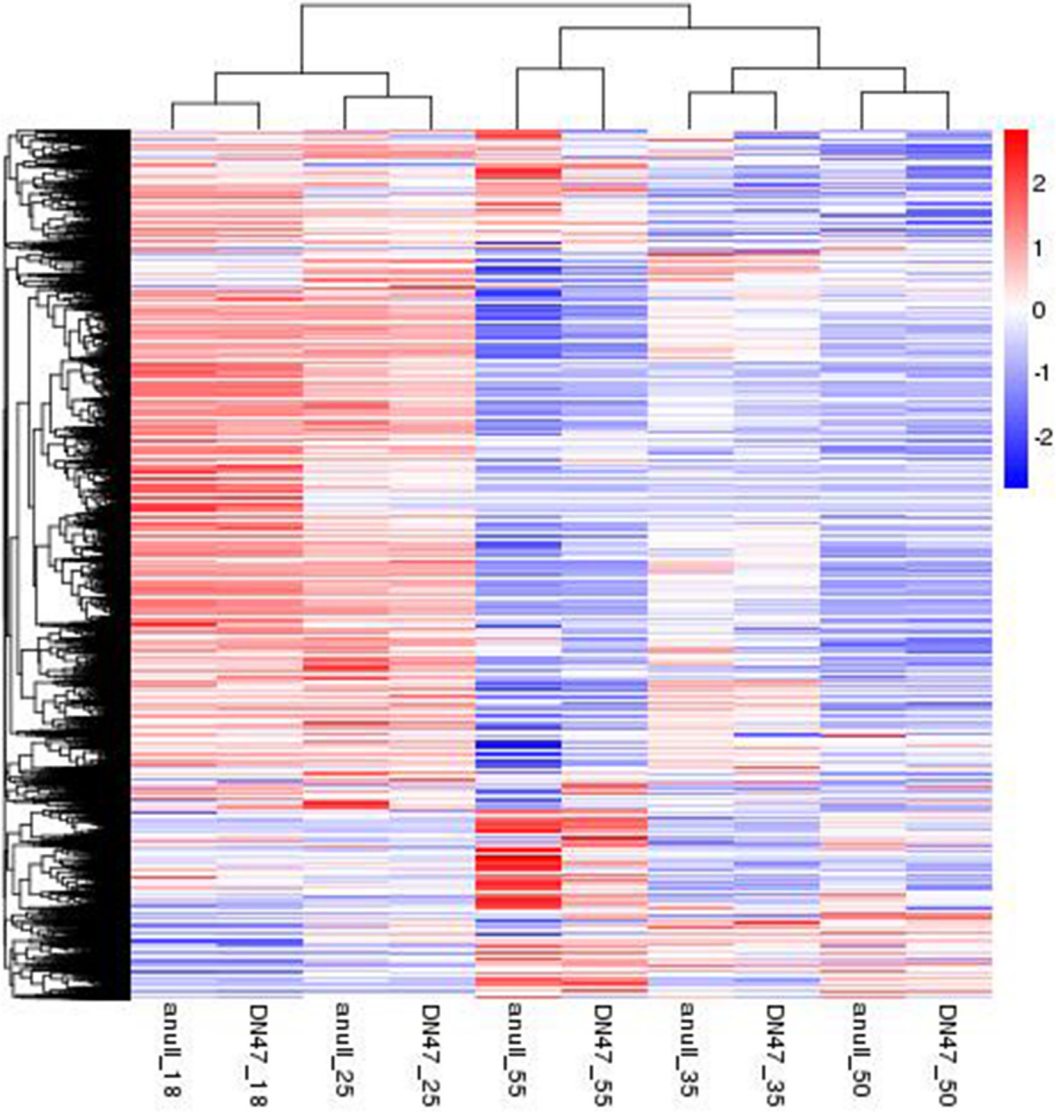
Cluster dendrogram of differentially expressed genes in *cgy-2* NIL and ‘DN47’ at five developmental stages (18, 25, 35, 50, and 55 days after flowering).

To determine whether these gene expression profiles correlated with development stages, the RNA-seq data of the *cgy-2-*NIL and DN47 were subjected to hierarchical clustering analysis using the ‘H-clust (1.10.1)’ function (Fig 5). The samples were clustered together based on genes that showed similar expression patterns. Genes expressed at the same stage both in *cgy-2-*NIL and DN47 were clustered together in all cases. The clusters of 18 DAF and 25 DAF seeds, and 35 DAF and 50 DAF seeds were very closely positioned, respectively. The 55 DAF cluster was closest to the 35 and 50 DAF clusters, and the 18 and 25 DAF clusters were farthest from the other three clusters (Fig 5). The greatest changes in gene expression were seen between the 25 and 55 DAF clusters. Notably, the developmental order was broken by 55 DAF; neighboring stages did not cluster together in the same order as development.

**Fig 6:**
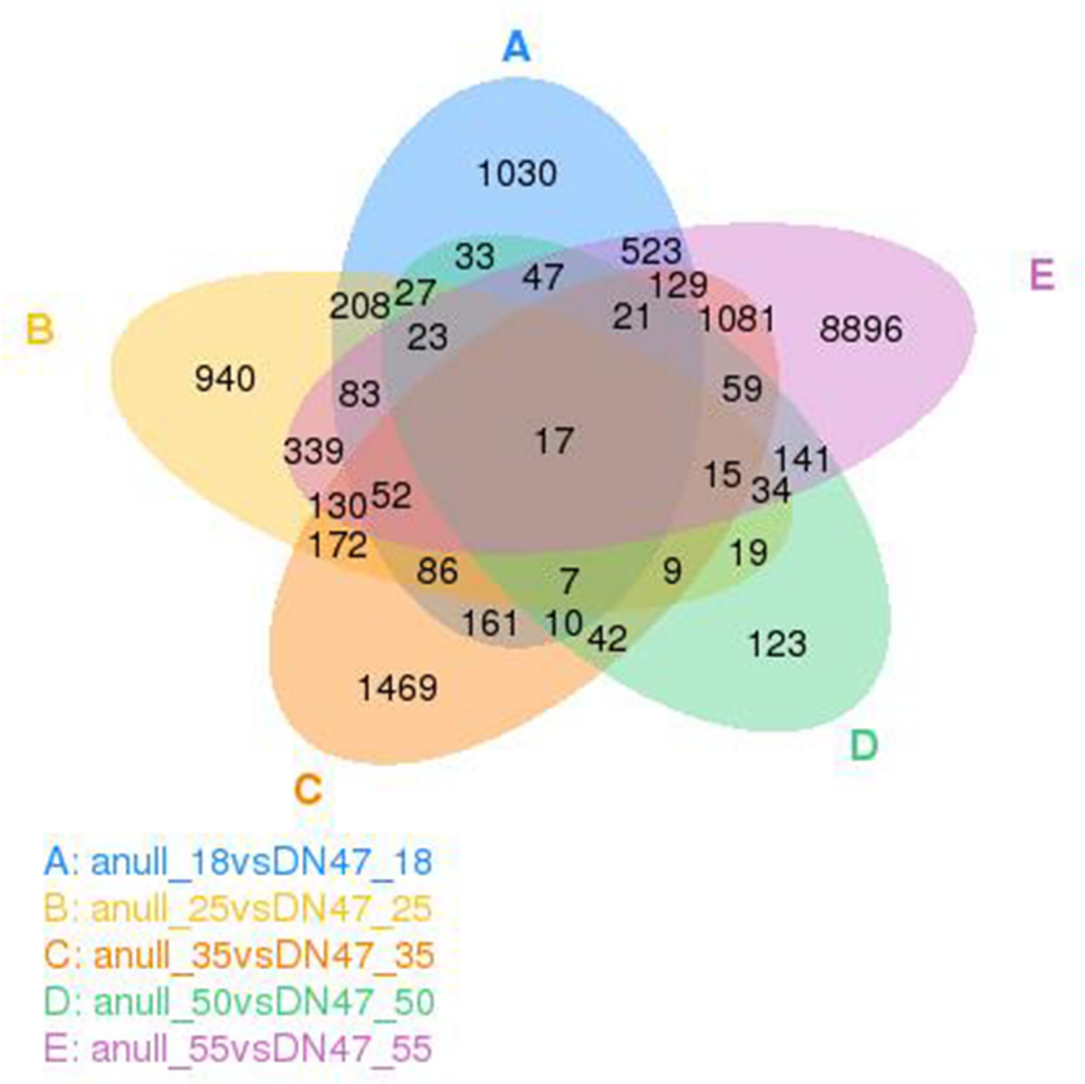
Venn diagram showing the overlap of identified differentially expressed genes (DEGs) between ‘cgy-2NIL’ and ‘DN47’ at 18, 25, 35, 50, and 55 days after flowering.

The comparison among different development stages between *cgy-2-*NIL and DN47 is shown in Fig 6. The majority of DEGs showed development-stage-specific expression. Seventeen DEGs were differentially expressed in all five stages. As shown in Table 3, among these 17 genes, only one signal transduction response regulator gene (Glyma11g15580) was upregulated during seed development. Five DEGs (Glyma02g41810, Glyma03g02370, Glyma04g41540, Glyma09g02600, and Glyma15g06160) were downregulated during seed development. The other 12 DEGs were differentially expressed among different developmental stages in both *cgy-2-*NIL and DN47, and exhibited two different expression patterns: one group, including two RNA recognition motif domain proteins (Glyma12g01350 and Glyma12g04710), one transcription factor MYC/MYB N-terminal (Glyma11g18290,) and one Ferritin-conserved site (Glyma01g31300) were upregulated in *cgy-2-*NIL only at 18 DAF, and were then downregulated in all subsequent stages. The other group, including Glyma02g04840, Glyma08g16310, Glyma11g25660, Glyma12g13920, Glyma20g16100, and Novel 100599, were upregulated in *cgy-2-*NIL at 25 DAF and 35 DAF, and downregulated at 18, 50, and 55 DAF.

**Table 3:** Seventeen genes with altered expression between ‘*cgy-2* NIL’ and ‘DN47’ across five developmental stages.

NO.	DEG ID	Log2-fold change					DESCRIPTION
		18 DAF	25 DAF	35 DAF	50 DAF	55 DAF	
1	GLYMA01G31300	0.791	−1.889	−1.969	−1.590	−3.658	Ferritin, conserved site||Ferritin||Ferritin- like di-iron domain||Ferritin-related||Ferritin/DPS protein domain||Ferritin/ribonucleotide reductase-like
2	GLYMA02G04840	−2.190	2.255	2.190	−4.465	−2.875	Protein of unknown function DUF241, plant
3	GLYMA02G41810	−1.321	−1.125	−0.759	−0.989	−1.103	Regulator of chromosome condensation, RCC1||Regulator of chromosome condensation 1/beta-lactamase-inhibitor protein II
4	GLYMA03G02370	−0.632	−0.646	−1.399	−1.554	−0.826	C2 calcium-dependent membrane targeting
5	GLYMA04G41540	−0.373	−0.886	−0.826	−0.837	−2.937	Glutamate synthase, NADH/NADPH, small subunit 1||"Glutamate synthase, alpha subunit, C-terminal"||"Glutamate synthase, central-C"
6	GLYMA08G16310	−0.751	2.011	1.320	−2.468	−1.264	-
7	GLYMA09G02600	−0.673	−1.084	−0.929	−2.871	−3.122	Haem peroxidase, plant/fungal/bacterial||Haem peroxidase||Peroxidases haem-ligand binding site||Plant peroxidase
8	GLYMA11G15580	0.603	0.716	0.740	1.607	1.567	Signal transduction response regulator, receiver domain||CheY-like superfamily
9	GLYMA11G18290	0.327	−0.524	−0.584	−1.232	−1.694	Myc-type, basic helix-loop-helix (bHLH) domain||Transcription factor MYC/MYB N-terminal
10	GLYMA11G25660	−3.218	1.637	1.709	−4.280	−3.175	EF-Hand 1, calcium-binding site||Calcium-binding EF-hand||EF-hand-like domain
11	GLYMA12G01350	0.272	−0.661	−0.793	−1.823	−2.004	Nucleotide-binding, alpha-beta plait||"Zinc finger, CCCH-type"||RNA recognition motif domain
12	GLYMA12G04701	0.348	−1.217	−0.846	−1.019	−1.247	Nucleotide-binding, alpha-beta plait||RNA recognition motif domain
13	GLYMA12G13920	−2.061	1.152	1.721	−1.622	−0.980	Glutaredoxin-like, plant II||Glutaredoxin||Thioredoxin-like fold
14	GLYMA15G06160	−0.371	−0.481	−0.660	−1.230	−0.774	Pseudouridine synthase, catalytic domain||Dyskerin-like||PUA-like domain||Pseudouridine synthase II
15	GLYMA16G07750	−1.014	−0.947	−0.793	−1.497	−1.077	-
16	GLYMA20G16100	−0.502	1.001	0.859	−1.231	−1.507	Development/cell death domain
17	Novel00599	−3.625	2.378	2.300	-	−2.038	

The top 20 genes that showed high-level differential expression related to the α-null mutation were ranked and are shown in Table 4. Glyma17g34220 (encoding alpha crystalline) and another six genes (Glyma13g11840, Glyma13g1189, Glyma13g11961, Glyma13g12033, Novel00815, and Novel01348), which were not annotated, were all downregulated at 18 DAF. Expression of Glyma13g11840 (no annotation) was downregulated by 12.16-fold, which was the most highly downregulated of all the DEGs identified in our data. Glyma20g28660 showed the highest differential expression related to α-null at 25, 35, and 50 DAF, and was downregulated by 9.32-fold at 25 DAF, 9.49-fold at 35 DAF, and 8.8-fold at 50 DAF. At 55 DAF, two genes (Glyma06g02690 and Glyma04g02660) encoding a Gibberellin-regulated protein and two genes (Glyma10g39760 and Glyma07g40110) encoding Concanavalin A-like lectins were downregulated by 9.03-fold and 7.91-fold, and by 8.47-fold and 6.93-fold, respectively. In addition, another four genes (Novel01985, Novel02415, Glyma15g10450, and Glyma16g03600) were upregulated at 55 DAF. Expression of Glyma15g10450, encoding a protein arginine N-methyltransferase, was upregulated by 8.05-fold; and Glyma16g03600, encoding an aminotransferase that takes part in cysteine and methionine metabolism, was upregulated by 7.56-fold. We hypothesized that these genes are putativelyα-null-related transcripts. Based on obtained DEGs information and bioinformatics, we will conduct further studies focused on gene function identification of the above-mentioned DEGs.

**Table 4:** The top 20 genes showing high-level differential expression related to the α-null mutation.

DEG ID	Stage(DAF)	Down/Up regulation		log2-fold change	KEGG	Description
GLYMA13G11840	18	Down		−12.162		-
GLYMA13G11895	18	Down		−8.0568		-
GLYMA13G11961	18	Down		−7.4505		
GLYMA13G12033	18	Down		−9.3913		-
GLYMA17G34220	18	Down		−8.145	Protein processing in endoplasmic reticulum	Alpha crystallin/Hsp20 domain||HSP20-like chaperone
Novel00815	18	Down		−7.0188		
Novel01348	18	Down		−7.7437		
GLYMA20G28660	25	Down		−9.32		
GLYMA20G28660	35	Down		−9.4945		Cupin 1||RmlC-like cupin domain||RmlC-like jelly roll fold
GLYMA20G28660	50	Down		−8.8458		Cupin 1||RmlC-like cupin domain||RmlC-like jelly roll fold
GLYMA15G10450	55		Up	8.0507		Protein arginine N-methyltransferase||S-adenosyl-L-methionine-dependent methyltransferase-like
GLYMA16G03600	55		Up	7.5585	Cysteine and methionine metabolism	Aminotransferase, class I/class II||"Aminotransferases, class I, pyridoxal-phosphate-binding site"||"Pyridoxal phosphate-dependent transferase, major region, subdomain 1"
Novel01985	55		Up	8.6001		
Novel02415	55		Up	8.0925		
GLYMA04G02660	55	Down		−7.914		Gibberellin-regulated protein
GLYMA06G02690	55	Down		−9.0289		Gibberellin-regulated protein
GLYMA07G39220	55	Down		−7.0642		Petal formation expressed
GLYMA07G40110	55	Down		−6.9337		Concanavalin A-like lectin/glucanase, subgroup||"Protein kinase, ATP binding site"||"Protein kinase, catalytic domain"||"Serine/threonine-protein kinase, active site"
GLYMA10G39760	55	Down		−8.4718		Concanavalin A-like lectin/glucanase, subgroup||"Glycoside hydrolase, family 16"||"Glycoside hydrolase, family 16, active site"||"Xyloglucan endo-transglycosylase, C-terminal"
GLYMA15G14675	55	Down		−7.4518		-

DEG = differentially expressed gene; DAF = days after flowering

### Functional annotation and pathway assignment

GO analysis was used to annotate the identified significant DEGs between *cgy-2-*NIL and DN47. Three main categories, biological process, molecular function, and cellular component, in developing seeds of *cgy-2-*NIL *vs*. DN47 at five stages (18, 25, 35, 50, and 55 DAF) are shown in Table 5. GO category enrichment analysis (P-value < 0.05) revealed different results in different stages. A similar GO category distribution pattern of transcripts was found at 18, 35, and 55 DAF (Table 5). For the biological process function, eight categories were identified, and the maximum number of DEGs was associated with the term ‘biosynthetic process’ at 18, 35, and 55 DAF. Eleven categories were identified as ‘cellular component’, and the terms ‘cellular component’ (18.89%, 19.95%, 22.26%), ‘cell’ (12.48%, 13.07%, 13.01%), and ‘cell part’ (12.48%, 13.07%, 13.01%) were the most abundant at 18, 35, and 55 DAF, respectively. In terms of molecular function, the most abundant DEGs were involved in structural molecular activity (64.41%, 58.10%, 58.36%) and structural constituents of ribosome (35.39%, 41.90%, 41.64%) at 18, 35, and 55 DAF, respectively. However, the GO category distributions of the transcripts at 25 and 50 DAF were quite different (Table 5). Through alignment with KEGG database, 6627 unigenes were annotated to 37 terms of GO classification at 25 DAF. Among these groups, ‘various biosynthetic process’ and ‘regulation of various metabolic process’ were dominant within the ‘biological process’ category. Only ‘apoplast’ was detected in the ‘cellular component’ category, and ‘ion binding’, ‘purine ribonucleoside triphosphate binding’, and ‘ATP binding’ were dominant in the molecular function category (Table 5) at 25 DAF. In addition, at 50 DAF, only seven terms belonging to ‘cellular component’ were annotated by GO category enrichment analysis.

**Table 5:** Summary of Gene Ontology (GO) terms for differentially expressed genes (DEGs) at different developmental stages (P < 0.05).

GO terms *cgy-2NILvs*. DN47	DESCRIPTION	Number of DEGs				
		18DAF	25DAF	35DAF	50DAF	55DAF
Biological process	biosynthetic process	597		760		2399
	organic substance biosynthetic process	577		727		2290
	cellular biosynthetic process	572		715		2232
	cellular macromolecule biosynthetic process	474		579		1752
	macromolecule biosynthetic process	476		582		1764
	gene expression	455		550		1650
	single-organism carbohydrate catabolic process	27		163		455
	translation	158		145		342
	organic cyclic compound biosynthetic process		283			
	RNA metabolic process		277			
	cellular nitrogen compound biosynthetic process		275			
	heterocycle biosynthetic process		274			
	aromatic compound biosynthetic process		269			
	nucleobase-containing compound biosynthetic process		256			
	RNA biosynthetic process		240			
	transcription, DNA-dependent		232			
	regulation of metabolic process		225			
	regulation of cellular metabolic process		214			
	regulation of macromolecule metabolic process		213			
	regulation of primary metabolic process		212			
	regulation of cellular biosynthetic process		210			
	regulation of biosynthetic process		210			
	regulation of macromolecule biosynthetic process		210			
	regulation of cellular biosynthetic process		210			
	regulation of nucleobase-containing compound metabolic process		209			
	regulation of nitrogen compound metabolic process		209			
	regulation of gene expression		208			
	regulation of transcription, DNA-dependent		204			
	regulation of RNA metabolic process		204			
	regulation of RNA biosynthetic process		204			
	cellular component movement		44			
	microtubule-based process		41			
	microtubule-based movement		33			
	cellular glucan metabolic process		22			
	glucan metabolic process		22			
Cellular component	cellular_component	902		1368		4164
	cell	596		896		2435
	cell part	596		896		2435
	intracellular	573		846		2305
	intracellular part	518		795		2143
	intracellular organelle	416		591		1654
	macromolecular complex	347		506		1238
	cytoplasm	294		399		1054
	cytoplasmic part	244		305		754
	ribonucleoprotein complex	153		139		296
	ribosome	137		117		232
	apoplast		12			
	cellular component organization or biogenesis				54	
	cellular component organization				49	
	cell morphogenesis				11	
	cellular component morphogenesis				11	
	anatomical structure morphogenesis				11	
	cellular developmental process				11	
	single-organism developmental process				11	
Molecular function	structural molecule activity	418		190		164
	structural constituent of ribosome	231		137		117
	ion binding		569			
	purine ribonucleoside triphosphate binding		310			
	ATP binding		296			
	cytoskeletal protein binding		59			
	tubulin binding		43			
	microtubule binding		42			
	motor activity		41			
	microtubule motor activity		33			
	xyloglucan:xyloglucosyl transferase activity		12			

DAF: days after flowering.

Pathway-based analysis is thought to provide a basic platform for the systematic analysis of DEGs involved in metabolic or signal transduction pathways. In this study, KEGG analyses were used to analyze gene function in terms of networks of gene products. Two types of DEGs, those up and downregulated at different development stages, were classified by KEGG, respectively (Table 6). In general, KEGG analysis assigned the DEGs (P < 0.05) of cgy-2NIL and DN47 to 16, 3, 9, 4, and 12 metabolic pathways (each of which contained 4–175 DEGs) at 18, 25, 35, 50, and 55 DAF, respectively (Table 6). At 18 DAF, we found several significant expression changes related to amino acid metabolism and fatty acid metabolism, including 13 genes involved in beta-alanine metabolism, six genes involved in histidine metabolism, 12 genes involved in arginine and proline metabolism, six genes involved in lysine degradation, and nine genes involved in fatty acid degradation, all of which were significantly upregulated. In addition, 41 genes involved in biosynthesis of amino acids showed significantly downregulated expression at 18 DAF. The majority of DEGs appeared to be related to ‘plant-pathogen interaction’ (32 genes, upregulated), ‘Ribosome biogenesis in eukaryotes’ (16 genes, downregulated), and ‘DNA replication’ (18 genes, downregulated) at 25 DAF. At 35 DAF, upregulated DEGs were assigned to ‘Ribosome’ and ‘photosynthesis’, while downregulated DEGs were assigned to seven KEGG pathways (protein processing in endoplasmic reticulum, ribosome biogenesis in eukaryotes, spliceosome, endocytosis, ABC transporters, RNA transport, and ubiquitin-mediated proteolysis). The DEGs identified at 55 DAF were assigned to 12 KEGG pathways. Pathways such as ribosome (145 genes, upregulated), biosynthesis of amino acids (114 genes, downregulated), and carbon metabolism (122 genes, downregulated) were highly represented.

**Table 6:** Kyoto Encyclopedia of Genes and Genomes (KEGG) assignment of differentially expressed genes (DEGs) identified in five developmental stages.

*cgy-2*NIL>DN47	Number of DEGs					Corrected P-value				
pathway	18 DAF	25 DAF	35 DAF	50 DAF	55 DAF	18 DAF	25 DAF	35DAF	50 DAF	55 DAF
Circadian rhythm—plant	19	-	-	-	26	1.51E-11		-	-	2.15E-02
beta-Alanine metabolism	13	-	-	-	-	1.28E-06	-	-	-	-
Fatty acid degradation	9	-	-	-	25	4.49E-04	-	-	-	2.15E-02
Histidine metabolism	6	-	-	-	-	5.34E-04	-	-	-	-
Arginine and proline metabolism	12	-	-	-	-	7.32E-04	-	-	-	-
Lysine degradation	6	-	-	-	-	2.26E-03	-	-	-	-
Plant-pathogen interaction	-	32	-	-	-	-	5.06E-09	-	-	-
Ribosome	-	-	96	-	145	-	-	4.64E-24	-	2.07E-09
Photosynthesis	-	-	41	5	-	-	-	3.07E-19	2.86E-02	-
Protein processing in endoplasmic reticulum	-	-	-	20	77	-	-	-	3.79E-10	1.89E-02
Photosynthesis—antenna proteins	-	-	-	4	-	-	-	-	3.46E-03	-
Spliceosome	-	-	-	-	81	-	-	-	-	4.27E-06
Peroxisome	-	-	-	-	40	-	-	-	-	2.00E-03
RNA transport	-	-	-	-	62	-	-	-	-	2.74E-03
Sulfur relay system	-	-	-	-	9	-	-	-	-	2.15E-02
Valine, leucine and isoleucine	-	-	-	-	23	-	-	-	-	2.40E-02
degradation										
Carotenoid biosynthesis	-	-	-	-	18	-	-	-	-	3.59E-02
***cgy-2*NIL<DN47**										
Ribosome	175	-	-	-	-	8.27E-59	-	-	-	-
Protein processing in endoplasmic reticulum	59	-	35	-	-	7.90E-08	-	1.56E-02	-	-
Ribosome biogenesis in eukaryotes	27	16	35	-	-	2.26E-04	4.96E-07	3.02E-10	-	-
DNA replication	18	18	-	12	-	5.37E-03	4.85E-11	-	2.18E-07	-
Carbon fixation in photosynthetic organisms	20	-	-	-	-	7.56E-03	-	-	-	-
Glycolysis / Gluconeogenesis	28	-	-	-	-	3.02E-02	-	-	-	-
Photosynthesis—antenna proteins	8	-	-	-	-	3.45E-02	-	-	-	-
Taurine and hypotaurine metabolism	7	-	-	-	-	4.26E-02	-	-	-	-
Plant-pathogen interaction	34	-	-	-	-	4.57E-02	-	-	-	-
Biosynthesis of amino acids	41	-	-	-	114	4.92E-02	-	-	-	3.09E-02
Spliceosome	-	-	55	-	-	-	-	1.02E-12	-	-
Endocytosis	-	-	29	-	-	-	-	3.12E-03	-	-
ABC transporters	-	-	10	-	-	-	-	8.22E-03	-	-
RNA transport	-	-	28	-	-	-	-	8.22E-03	-	-
Ubiquitin-mediated proteolysis	-	-	22	-	-	-	-	4.35E-02	-	-
Carbon metabolism	-	-	-	-	122	-	-	-	-	1.18E-02

DAF: days after flowering.

### Transcription factors (TFs) affected by the ‘α-null’ mutation

TFs are important proteins that control the flow of genetic information from DNA to RNA, and ultimately affect the growth and physiology of the plant. In the present study, 74 TFs were differentially expressed between *cgy-2-*NIL and DN47, when a fold change ≥ 1 and P < 0.05 were used as cutoff values (Table 7). These genes were divided into different classes, as shown in Table 7. These TFs included BREVIS RADIX, GRAS, jumonji, GATA, SBP-box, and TCP. The most abundant TF group was GRAS. Among all the identified GRAS TFs, four (Glyma06G41500, Glyma07G39650, Glyma12G34420, and Glyma13G36120) were downregulated at 18 DAF in *cgy-2-*NIL; however, nine GRAS TFs were significantly upregulated at 25 DAF in *cgy-2-*NIL. Only one GRAS TF, Glyma12G34420, was identified as upregulated at 35 DAF. Eighteen GRAS TFs that were differentially expressed at 55 DAF displayed different expression patterns: 11 were downregulated in *cgy-2-*NIL, while seven were upregulated. The 55 DAF stage was characterized by the highest number of differentially expressed TFs in *cgy-2-*NIL compared with DN47, and there were more downregulated than upregulated TFs (39 *vs*. 20). Notably, 13 MADS-box TFs were all downregulated at 55 DAF. By contrast, the fewest number of TF genes was found at 50 DAF; only one upregulated TF, TCP (Glyma03G02090) and one downregulated TF, GATA (Glyma04G01090) were identified. In addition, we observed that in the 35 DAF whole seed, five groups of TFs were differentially expressed, including BREVIS RADIX (Glyma09G34601 and Glyma16G17590), GRAS (Glyma12G34420), jumonji (Glyma09G34040) and SBP-box (Glyma04G37391).

**Table 7:** Summary and annotation of transcription factors (TFs) selected using RPKM analysis of RNA-seq data.

Category of TF	Gene ID	Gene Annotation	RPKM									
			18 DAF		25 DAF		35 DAF		50 DAF		55 DAF	
			α-null	DN47	α-null	DN47	α-null	DN47	α-null	DN47	α-null	DN47
BREVIS RADIX	GLYMA09G07930	Transcription factor BREVIS RADIX									1.69	0.68
	GLYMA09G34601						8.68	15.39				
	GLYMA16G17590						1.20	2.27				
GRAS	GLYMA01G18040	Transcription factor GRAS									0.98	3.89
	GLYMA01G33270										0.61	1.54
	GLYMA01G40180										2.02	1.03
	GLYMA01G43620										2.02	1.03
	GLYMA02G46730				3.63	1.34						
	GLYMA03G03760										2.36	5.89
	GLYMA04G42090										0.81	2.61
	GLYMA05G03490				17.29	7.03						
	GLYMA06G11610										1.87	0.73
	GLYMA06G41500		0.59	1.61	3.37	1.28						
	GLYMA07G39650		9.40	25.91	30.06	13.50						
	GLYMA08G43780										0.75	4.82
	GLYMA09G01440				8.48	2.97						
	GLYMA09G04110										0.12	1.41
	GLYMA10G37640										8.87	4.01
	GLYMA11G01850										0.32	1.00
	GLYMA11G14710										0.45	1.56
	GLYMA11G17490										1.37	3.51
	GLYMA12G02060										4.05	1.82
	GLYMA12G06670										0.48	1.29
	GLYMA12G16750				2.80	0.71						
	GLYMA12G34420		4.02	12.13			18.78	5.28			97.64	24.52
	GLYMA13G09220											
	GLYMA13G36120		11.15	26.56	46.00	20.59						
	GLYMA15G12320				3.05	1.35						
	GLYMA17G14030				20.78	8.61						
	GLYMA18G45220										1.09	4.16
	GLYMA20G30150										21.61	7.71
IIc	GLYMA14G24776	Transcription factor IIIC, 90kDa subunit, N-terminal									6.27	2.53
	GLYMA19G40560	Transcription factor, WRKY group IIc									0.97	2.62
jumonji, JmjN	GLYMA10G35350	Transcription factor jumonji, JmjN									7.92	4.35
	GLYMA09G34040	Transcription factor jumonji, JmjN					2.64	4.91				
MYC/MYB	GLYMA06G04550	Transcription factor MYC/MYB N-terminal									0.21	2.21
	GLYMA17G31537										4.00	1.87
TFIIE	GLYMA05G38060	Transcription factor TFIIE beta subunit,									22.66	11.71
	GLYMA05G07910	Transcription factor TFIIE, alpha subunit									8.06	3.81
GATA	GLYMA04G01090	Transcription factor, GATA, plant			16.26	7.06			0.33	1.86		
	GLYMA10G35470										0.84	2.57
	GLYMA16G27171										0.74	5.79
K-box MADS-box	GLYMA01G08130	Transcription factor, K-box									1.64	3.87
	GLYMA02G13401										0.76	1.87
	GLYMA04G43640										7.40	20.22
	GLYMA05G07286										0.12	1.98
	GLYMA06G48270										8.24	24.63
	GLYMA08G42300										15.20	39.28
	GLYMA11G16105										0.16	2.52
	GLYMA11G36890										0.73	2.89
	GLYMA13G06730										5.30	17.27
	GLYMA13G29510										0.81	2.07
	GLYMA14G03100										2.01	5.78
	GLYMA18G12590										1.36	5.20
	GLYMA19G04320										16.72	53.88
NFYB/HAP3CBF/NF-Y	GLYMA09G01650	Transcription factor, NFYB/HAP3Transcription factor, CBF/NF-Y									0.62	2.99
	GLYMA10G05606										20.68	9.31
	GLYMA17G00950										0.61	4.26
SBP-box	GLYMA02G13371	Transcription factor, SBP-box									0.36	1.07
	GLYMA03G27195										0.36	1.07
	GLYMA04G37391						0.25	1.48				
TCP	GLYMA03G02090	Transcription factor, TCP							1.23	0.24	0.06	0.40
	GLYMA05G00300										1.74	4.07
	GLYMA05G01131										2.81	0.79
	GLYMA10G06515										5.30	2.02
	GLYMA12G28970										0.46	2.65
	GLYMA12G35720										2.40	5.57
	GLYMA18G50371										3.55	1.60
DELLA GRAS	GLYMA11G33720	Transcription factor DELLA, N-terminal									4.34	17.33
	GLYMA18G04500										4.45	14.14
Others	GLYMA11G14450	Transcription factor IIA, alpha/beta subunit, N-terminal									46.38	23.63
	GLYMA03G28000	Transcription factor IIS, N-terminal									14.76	6.34
	GLYMA19G31830	Transcription factor TFIIB, conserved site									3.55	1.00
	GLYMA13G07720	Transcription factor, MADS-box									0.26	1.02

RPKM: reads per kilobase of transcript per million reads mapped; DAF: days after flowering.

### Gene models annotated as Cupin proteins

Previous studies have characterized the cupins as important allergens in peanuts and soybeans [38–40]. The majority of cupin allergens belong to either the 11S legumin-like or the 7S vicilin-like seed storage globulin families. To better characterize the effect of α-null mutations on the differential expression of allergen genes, particular attention was paid to the cupin protein family in *cgy-2-*NIL. In the present study, 18 genes in Table 8 are annotated as encoding cupin proteins. In general, these genes showed peak expression (in RPKM) at 35 or 50 DAF, with RPKMs ranging from 0 to 52124.19. Most of these cupin genes were downregulated in *cgy-2-*NIL compared with DN47 throughout the five development stages.

**Table 8:** Summary of differentially expressed genes (DEGs) annotated as Cupin proteins.

CUPIN DEGs				RPKM												
		18 DAF		Log2 fold change	25 DAF		Log2 fold change	35 DAF		Log2 fold change	50 DAF		Log2 fold change	55 DAF		Log2 fold change
GENE ID	Homologs	*cgy*-2NIL	DN47		*cgy*-2NIL	DN47		*cgy*-2NIL	DN47		*cgy*-2NIL	DN47		*cgy*-2NIL	DN47	
GLYMA10G39150	7S(αˊ-subunit)	1.1597	1.7119	-0.6044	3398.4610	7830.8560	-1.3112	18998.7400	18363.3300	-0.0982	12163.3200	12909.6100	-0.1835	1283.1920	4885.0200	-1.7435
GLYMA20G28650	7S(α-subunit)	0.0000	0.0075	#	2.0333	51.8679	-4.7708	6.1619	90.2581	-4.0533	4.6006	37.4387	-3.1152	0.1290	9.2461	-5.9973
GLYMA20G28660	7S(α-subunit)	0.0000	0.0000	#	0.0265	15.7801	-9.3200	0.1392	91.3088	-9.4945	0.0825	35.4523	-8.8458	0.0000	1.2569	#
GLYMA20G28460	7S(β-subunit)	0.0000	0.0000	#	0.0904	0.5778	-2.8160	291.5820	160.9376	0.6753	1082.1720	718.6699	0.5156	113.0759	508.6404	-1.9759
GLYMA20G28640	7S(β-subunit)	0.0786	0.0721	0.0725	0.2241	3.2988	-3.9921	500.7281	371.2632	0.2595	1258.4940	1091.4660	0.1226	158.1022	606.8583	-1.7488
GLYMA10G03390	7S	1.1380	1.6587	-0.5859	1095.8900	2584.6960	-1.3444	5619.9860	6335.0460	-0.3204	4041.8420	4589.9470	-0.2679	601.9122	3318.1880	-2.2761
GLYMA02G16440	7S	0.7838	0.6071	0.3265	73.8799	260.0706	-1.9291	1747.4270	1922.5170	-0.2792	1811.1770	1356.9570	0.3192	645.7540	586.5564	0.3266
GLYMA10G39170	7S	0.3624	0.3954	-0.1708	47.1786	122.0998	-1.4848	637.6302	734.9372	-0.3510	1636.7760	1094.2250	0.4743	1568.4520	1781.4970	0.0035
GLYMA03G32030	11S(Gy1)	0.0090	0.0887	-3.3269	1519.8480	6668.6300	-2.2490	26382.6200	35866.6200	-0.5813	46783.7600	52124.1900	-0.2404	1808.5410	16498.6800	-2.9965
GLYMA03G32020	11S(Gy2)	0.0140	0.0342	-1.3358	734.0615	3416.8350	-2.3344	16533.9400	21521.1000	-0.5223	33946.0400	36257.4200	-0.1794	1675.7170	11668.7100	-2.6104
GLYMA19G34780	11S(Gy3)	0.0185	0.0341	-0.9092	20.3979	38.4533	-1.0454	4697.7480	4131.7350	0.0606	6783.2350	6454.2300	-0.0397	26.6057	260.1870	-3.1019
GLYMA10G04280	11S(Gy4)	0.0000	0.0506	#	188.9422	1070.9180	-2.6277	14273.1500	18260.9700	-0.4876	18121.2500	21300.1900	-0.3150	429.2719	4996.3280	-3.3479
GLYMA13G18450	11S(Gy5)	0.0000	0.0253	#	93.8437	526.5713	-2.6155	12032.5500	14774.3600	-0.4303	20977.4600	22500.2000	-0.1946	412.3518	4566.2670	-3.2790
GLYMA19G34770	11s(Gy7)	0.2984	0.1888	0.6230	1.8343	1.5277	0.1746	6.6207	5.0027	0.2368	13.16456	6.525997	0.9375	20.80936	17.72077	0.4226
GLYMA08G13440	11S	1.5047	1.5675	-0.1020	6.7538	7.3609	-0.2235	33.4617	43.2815	-0.5304	9.6622	11.0915	-0.2635	0.6632	2.7409	-0.7581
GLYMA15G04710	11S	120.6579	102.0619	0.1985	75.7392	75.3835	-0.0973	62.8506	43.7549	0.3594	21.5767	19.7137	0.0468	3.9780	7.8482	-0.4931
GLYMA16G00980		0.4581	0.2641	0.7496	1.6147	1.3215	0.1921	5.8274	2.7490	0.9221	0.0588	0.1320	-1.2224	0.0000	0.0602	#
GLYMA10G39161		0.0971	0.0990	-0.0746	0.1682	0.3765	-1.2215	2.4629	0.5772	1.9352	0.7714	0.0000	#	0.0849	0.0495	0.9501

Shadowing indicates a significant change in gene expression between ‘*cgy-2* NIL’ and ‘DN47’. RPKM: reads per kilobase of transcript per million reads mapped; DAF: days after flowering. #: One of the data is zero, cannot use multiple expression.

Among the 18 cupin genes, five belong to the β-conglycinin subunit gene family, including Glyma10g39150 encoding the α′-subunit, whereas Glyma20g28460 and Glyma20g28640 encode the β-subunit, and Glyma20g28650 and Glyma20g28660 encode the α-subunit [41] (http://www.Phytozome.net/soybean) (Table 8). The expression of α′-subunit gene (Glyma10g39150) was detected at 18 DAF, which is earlier than both the α-and β-subunit genes and peaked at 35 DAF. Its RPKM level was much higher than both the α- and β-subunit genes during the five developmental stages. Glyma10g39150 (α′-gene) showed downregulated expression throughout the five development stages in *cgy-2-*NIL (Table 8). Notably, the α-null mutation was associated with significantly reduced expression of both α-subunit genes, Glyma20G28650 and Glyma20G28660, in proportional amounts. The expression level of Glyma20g28650 was consistently higher than Glyma20G28660 from 25 to 55 DAF in *cgy-2-*NIL. The two genes showed almost no expression at 18 DAF, and began to be highly expressed at 25 DAF, showing peak expression at 35 DAF, which then declined until 55 DAF in DN47. Similar expression patterns of Glyma20G28650 and Glyma20G28660 were found in *cgy-2-*NIL; however, the level of expression of Glyma20G28650 was much lower in *cgy-2-*NIL than in DN47 at the same stage, while Glyma20G28660 was barely expressed throughout the five developmental stages in *cgy-2-*NIL. The two β-subunit genes of β-conglycinin, Glyma20g28460 and Glyma20g28640, also showed different expression levels between *cgy-2-*NIL and DN47. The expression levels of Glyma20g28460 and Glyma20g28640 in *cgy-2-*NIL were lower than those in DN47 at 25 DAF (by 2.8160-fold and 3.9921-fold, respectively), and at 55DAF (by 1.9759- and 1.7488-fold, respectively); However, in the other stages (35 and 50 DAF), the two β-subunit genes in *cgy-2-*NIL showed higher expression (Log2 fold change from 0.1226 to 0.6753) than in DN47.

In addition, another six differentially expressed gene IDs matched glycinin subunit genes *Gy1-7* (Glyma03g32030 to *Gy1*, Glyma03g32020 to *Gy2*; Glyma19g34780 to *Gy3*; Glyma10g04280 to *Gy4*; Glyma13g18450 to *Gy5*; Glyma19g34770 to *Gy7*) [42–44]. Among these six genes, the expressions of *Gy1*, *Gy2*, *Gy4*, and *Gy5* in *cgy-2*-NIL were all lower than that in DN47 throughout five developing stages, and these genes showed a similar pattern of expression, i.e., starting at about 18–25 DAF, showing a peak in RPKM at 50 DAF, and then declining rapidly thereafter (Table 8). The expression level of *Gy3* in *cgy-2-NIL* was lower than that in DN47 at 18, 25, and 55 DAF, but higher at 35 and 50 DAF. *Gy7* expression was drastically lower than the other five glycinin genes, both in *cgy-2-NIL* and DN47 from 25 DAF to 55 DAF. Furthermore, *cgy-2-NIL* had higher expression levels of *Gy7* than that in DN47 throughout the five stages examined in the present study.

### qRT-PCR validation of differential gene expression in *cgy-2-*NIL and DN47

We used qRT-PCR to validate selected DEGs identified from the RNA-seq data. Six DEGs (GLYMA06G02690, GLYMA07G39650, GLYMA11G33720, GLYMA12G34420, GLYMA18G04500 and GLYMA20G28530) that were differentially expressed in all five stages were selected, which included up- and downregulated genes between *cgy-2-*NIL and DN47. The relative expression changes of the selected genes are shown in Fig 3: a positive correlation (R^2^ = 0.6069) between the RNA-seq data and qRT-PCR data was detected (Fig 3B). All six selected genes showed consistent up or downregulated expression patterns throughout all five detected stages, respectively, confirming the RNA-seq data (Fig 3C).

## Discussion

The α-subunit is one of the major components of soybean seed storage proteins; therefore, the complete deficiency of the α-subunit should change the gene’ expression profiles and metabolic pathways during seed maturation. NILs are valuable genetic resources to identify genomic regions and alleles responsible for trait variation [45], and are also particularly suitable for genetic analyses of transcriptome and proteome variations. To further understand the potential mechanisms involved in the regulation of the α-null mutation, we have used RNA-seq to investigate global gene expression changes over five stages of soybean cotyledon development in seeds of α-subunit-deficient NIL lines (*cgy-2-*NIL). We have identified several critical genes that were possibly associated with the α-null mutation. Only Glyma20G28660 was annotated as the α-subunit gene of β-conglycinin, and appeared to be significantly downregulated throughout the three green stages (25, 35, and 50 DAF) of development studied here. Surprisingly, at 55 DAF (the desiccating stage of development), the number of DEGs was the highest. This observation is consistent with the results of a previous report [26], in which many genes showed peak expression at the latter stages of seed maturation. These genes were annotated as being TFs or related to protein degradation [26]. Our analyses have also resulted in the identification of interesting late expressed DEGs (at 55 DAF). In particular, Glyma16G03600, which is involved in cysteine and methionine metabolism, was upregulated by 7.56-fold in ‘*cgy-2-*NIL’, and Glyma04G02660 and Glyma06G02690, which were annotated as gibberellin-regulated protein genes. We also predicted many novel candidate genes that were associated with the α-null mutation, which provide a strong basis for future research on determining the molecular mechanism of α-subunit-null deficiency. To determine whether the differential expression of genes such as Glyma16G03600, Glyma04G02660, and Glyma06G02690 have a direct relation to α-subunit-null mutation, the function of these DEGS will be studied by RNA interference or by overexpression in transgenic plants in the future. This could lead to a better understanding of the molecular regulation of storage protein subunit accumulation in the α-null mutant.

The cupins are a large superfamily, named on the basis of a conserved ‘double-stranded *β*-helix’ barrel-like structure (‘*cupa*’ means ‘small barrel’ in Latin). The majority of cupin allergens were originally discovered using a conserved motif found within the 7S vicilin-like or 11S legumin-like seed storage globulin families from higher plants [46]. The cupin superfamily of proteins possesses remarkable functional diversity, with representatives found in the Archaea, Eubacteria, and Eukaryota [47–49]. Previous studies characterized the majority of cupin allergens as belonging to either the 11S legumin-like or 7S vicilin-like seed storage globulin families. In our study, 16 storage protein subunit genes, eight 7S-related subunits, and eight 11S-related subunits were included in the cupin group (Table 8).

Soybean seeds contain between 35 and 45% protein on a dry weight basis, of which about 70% consists of the two major storage proteins, 7S globulin (β-conglycinin) and 11S (glycinin). Development changes in the synthesis of β-conglycinin and glycinin have been described previously [50,25,26]. In the present study, the expression of various subunit gens of β-conglycinin and glycinin in both *cgy-2*-NIL and DN47 showed similar developmental expression patterns: they presented a bell-shaped pattern of expression that started at 18–25 DAF, reached a maximum at 35 DAF or 50 DAF, and declined rapidly thereafter. The α′-and α-subunit genes of β-conglycinin reached their expression peaks (at 35 DAF) before the β-subunit genes of β-conglycinin and five glycinin, *Gy1*–*Gy5* subunit genes (at 50 DAF). These results were similar to earlier observations [50, 25, 26]. However, the expression levels of 18 cupin genes in *cgy-2*-NIL were significantly different to those in DN47. The α-null mutation caused almost all the β-conglycinin (α′-, α-, and β-subunit) genes and glycinin (Gy1-, Gy2-, Gy3-, Gy4-, Gy5-, -subunits) genes to show downregulated expression in at least two stages of development studied here. The expressions of various β-conglycinin and glycinin subunit genes were regulated coordinately in the *cgy-2*-NIL, which might be responsible for the altered amino acid composition and improved protein quality.

Previous analysis of β-conglycinin-deficient lines revealed that the loss of β-conglycinin was compensated for by an increase in the abundance of glycinin [1]. Glycinin, an 11S globulin, is the predominant seed storage protein in soybean, and makes an important contribution to the nutritional quality of soy protein. In the present study, compared with DN47, the α-null mutation caused glycinin *Gy3* to be upregulated at 35 DAF and *Gy7* was upregulated throughout all five stages. To date, five glycinin genes, *Gy1*–*Gy5* have been described in detail. *Gy4* and *Gy5* encode proteins that have lower concentration of sulfur amino acids than the proteins derived from *Gy1*, *Gy2*, and *Gy3* [51]. Furthermore, Belinson et al. [44] identified and mapped a new functional glycinin gene, *Gy7*, which encodes the sixth glycinin subunit Gy7. Their data revealed that the steady-state amount of mRNA encoding Gy7 at seed mid-maturation is an order of magnitude less than the mRNA encoding the five other glycinin subunits [44]. Similar results were obtained in our study, which further confirmed that the *GY7* gene has a lower expression level than the five other glycinin subunits from 25 to 55 DAF, both in *cgy-2-*NIL and DN47. To date, little is known about the effect of the Gy7 subunit on protein nutritional quality, tofu-making quality, and its health benefits. Different from the other five glycinin genes, *GY7* expression in *cgy-2-*NIL slightly exceeded that of DN47 throughout the five stages identified in the present study, and showed a unique developmental expression pattern in both *cgy-2-*NIL and DN47, i.e., increased from the 18 DAF until reaching a peak at 55 DAF. The upregulated expressions of *Gy3* and *Gy7* might, at least in part, contribute to the modified final seed protein content in *cgy-2*-NIL.

## Conclusions

We present an overview of genes whose expression was affected by the ‘α-null’ mutation in soybeans. A number of soybean genes with annotations related to cupin allergen proteins, transcription factors, and other processes were differentially expressed in *cgy-2-*NIL. Some of these genes may be candidates for hypoallergenic soybean breeding. The *cgy-2* allele in the homozygous form modified the expression level of various β-conglycinin and glycinin cupin-family-genes. The desiccating stage of development (55DAF), is a critical period of differential gene expression. Our findings will help provide a detailed understanding of the α-subunit-null mechanism. In addition, the *cgy-2* allele was validated as an effective and useful allele for soybean breeding programs that aim to modify protein quality and reduce allergenicity.

## References

[1] Ogawa T, Tayama E, Kitamura K, Kaizuma N. Genetic improvement of seed storage proteins using three variant alleles of 7S globulin subunits in soybean (*Glycine max L*.). Jpn J Breed. 1989;39: 137–147.

[2] Takahashi M, Uematsu Y, Kashiwaba K, Yagasaki K, Hajika M, Matsunaga R, et al Accumulations of high levels of free amino acids in soybean seeds through integration of mutations conferring seed protein deficiency. Planta. 2003;217: 577–586. 12684787 10.1007/s00425-003-1026-3

[3] Kita Y, Nakamoto Y, Takahashi M, Kitamura K, Wakasa K, Ishimoto M. Manipulation of amino acid composition in soybean seeds by the combination of deregulated tryptophan biosynthesis and storage protein deficiency. Plant Cell Rep. 2010;29: 87–95. 10.1007/s00299-009-0800-5 19943163

[4] Harada K, Hayashi M, Tsubokura Y. Genetic variation of globulin composition in Soybean Seeds. Agricultural Research Updates. 2013; 101–116.

[5] Ogawa A, Samoto M, Takahashi K. Soybean allergens and hypoallergenic soybean products. J Nutr Sci Vitaminol. 2000;46: 271–279. 11227798 10.3177/jnsv.46.271

[6] Takahashi K, Shimada S, Shimada H, Takada Y, Sakai T, Kono Y, et al A new soybean cultivar “Yumeminori” with low allergenicity and high content of 11S globulin. Bull Natl Agric Res Cent Tohoku Reg. 2004;102: 23–39.

[7] Kitamura K, Kaizuma N. Mutant strains with low level of subunits of 7S globulin in soybean (*Glycine max Merillr*.) seeds. Jpn J Breed. 1981;31: 353–359.

[8] Harada K, Toyokawa Y, Kitamura K. Genetic analysis of the most acidic 11S globulin subunit and related characters in soybean seeds. Jpn J Breed. 1983;33: 23–30.

[9] Odanaka H, Kaizuma N. Mutants on soybean storage proteins induced with γ-ray irradiation. Jpn J Breed. 1989;39(Suppl.): 430–431.

[10] Takahashi K, Banba H, Kikuchi A, Ito M, Nakamura S. An induced mutant line lacking the α-subunit of β-conglycinin in soybean (*Glycine max (L*.*) Merrill*). Breed Sci. 1994;46: 65–66.

[11] Hajika M, Takahashi M, Sakai S, Igita M. A new genotype of 7S globulin (β-conglycinin) detected in wild soybean (*Glycine soja Sieb*. *EtZucc*.). Breed Sci. 1996;46: 385–386.

[12] Song B, Shen LW, Wei XS, Guo BW, Tuo Y, Tian FD, Li WB, Liu SS. Marker-assisted backcrossing of a null allele of the α-subunit of soybean (*Glycine max*) β-conglycinin with a Chinese soybean cultivar. Plant Breeding. 2014;133(5): 638–648.

[13] Hajika M, Sakai S, Matsunaga R. Dominant inheritance of a trait lacking b-conglycinin detected in a wild soybean line. Breed Sci. 1998;48: 383–386.

[14] Hajika M, Takahashi K, Yamada T, Komaki K, Takada Y, Shimada H, et al Development of a new soybean cultivar for soymilk, “Nagomimaru”. Crop Sci. 2009;10: 1–20.

[15] Thanh VH, Shibasaki K. Major proteins of soybean seeds: subunit structure of beta-conglycinin. J Agric Food Chem. 1978;26: 692–695.

[16] Koshiyama I. Chemical and physical protein in soybean globulins. Cereal Chem. 1968;45: 394–404.

[17] Ogawa T, Bando N, Tsuji H, Nishikawa K, Kitamura K. Alpha-subunit of beta-conglycinin, an allergenic protein recognized by IgE antibodies of soybean-sensitive patients with atopic dermatitis. Biosci Biotechnol Biochem. 1995;59: 831–833. 7787297 10.1271/bbb.59.831

[18] Fu CJ, Jez JM, Kerley MS, Allee GL, Krishnan HB. Identification, characterization, epitope mapping, and three dimensional modeling of the alpha-subunit of beta-conglycinin of soybean, a potential allergen for young pigs. J Agric Food Chem. 2007;55: 4014–4020. 17439152 10.1021/jf070211o

[19] Holzhauser T, Wackermann O, Ballmer-Weber BK, Bindslev-Jensen C, Scibilia J, Perono-Garoffo L, et al Soybean (*Glycine max*.) allergy in Europe: Gly m 5 (beta-conglycinin) and Gly m 6 (glycinin) are potential diagnostic markers for severe allergic reactions to soy. J Allergy Clin Immunol. 2009;123: 452–458. 10.1016/j.jaci.2008.09.034 18996574

[20] Krishnan HB, Kim WS, Jang S, Kerley MS. All three subunits of soybean beta-conglycinin are potential food allergens. J Agric Food Chem. 2009;57: 938–943. 10.1021/jf802451g 19138084

[21] Tsukada Y, Kitamura K, Harada K, Kaizuma N. Genetic analysis of subunits of two major storage protein (β-conglycinin and glycinin) in soybean seeds. Japan. J. Breed. 1986;36: 390–400.

[22] Kitamura K, Davis CS, Nielsen NC. Inheritance of alleles of Cgy1 and Cgy4 storage protein genes in soybean. Theor Appl Genet. 1984; 68: 253–257. 10.1007/BF00266899 24259062

[23] Takahashi K, Mizuno Y, Yunoto S, Kitamura K, Nakamura S. Inheritance of α subunit deficiency of β-conglycinin in soybean (*Glycine max L*. *MERRILL*) line induced by γ-ray irradiation. Breeding Science. 1996;46: 251–255.

[24] Narikawa T, Tamura T, Yagasaki K, Terauchi K, Sanmiya K, Ishimaru Y, et al Expression of the stress-related genes for glutathione S-transferase and ascorbate peroxidase in the most-glycinin-deficiency soybean cultivar Tousan205 during seed maturation. Biosci Biotechnol Biochem. 2010;74(9): 1976–1979. 20834138 10.1271/bbb.100479

[25] Asakura T, Tamura T, Terauchi K, Narikawa T, Yagasaki K, Ishimaru Y, et al Global gene expression profiles in development soybean seeds. Plant Physiol Biochem. 2012;52: 147–153. 10.1016/j.plaphy.2011.12.007 22245912

[26] Jones SI, Gonzalex DO, Vodkin LO. Flux of transcript patterns during soybean seed development. BMC Genomics. 2010;11: 136 10.1186/1471-2164-11-136 20181280 PMC2846912

[27] Jones SI, Vodkin LO. Using RNA-Seq to profile soybean seed development from fertilization to maturity. PLOS ONE. 2013;8: 1–12. 10.1371/journal.pone.0059270 PMC3598657 23555009

[28] Laemmli UK. Cleavage of structural proteins during the assembly of the head bacteriophage T4. Nature. 1970;277: 680–685. 10.1038/227680a0 5432063

[29] FAO/WHO. Protein quality evaluation. Report of the joint FAO/WHO expert consultation Rome: FAO Food and Nutrition; 1991 pp. 51.

[30] Mitchell HH. The wastage of nutrients in metabolism: proteins and amino acids. Comparative nutrition of man and domestic animals New York: Academic Press; 1964 pp. 567–659.

[31] Hidvegi M. Bekes F. Mathematical of protein quality from amino acid composition. In: Lasztity R, Hidvegi M, editors. Processing of the International Association of the Cereal Chemistry Symposium. Budapest: Akademiaikiado; 1984. pp. 205–286.

[32] Oser BL. An integrated essential amino acid index for predicting the biological value of proteins In: Albanese AA, editor. Protein and amino acid nutrition. New York: Academic Press; 1959 pp. 281–295.

[33] Guo QQ, Ma. XJ. Wei SG. Bai LH. Fu JE. Dong SK, Liu LJ, Zu W. Isolation of RNA from uncaria with medicinal plant. Crop. 2013;5: 80–83.

[34] Mortazavi A, Williams BA, et al Mapping and quantifying mammalian transcriptomes by RNA-Seq. Nature Methods. 2008;5(7): 621–628. 10.1038/nmeth.1226 18516045 PMC13303166

[35] Anders S, Huber W. Differential expression analysis for sequence count data. Genome Biol. (DESeq). 2010;11(10): R106 10.1186/gb-2010-11-10-r106 PMC3218662 20979621

[36] Young MD, Wakefield MJ, Smyth GK, Oshlack A. Gene ontology analysis for RNA-seq: accounting for selection bias. Genome Biology. (GOseq). 2010;11(2): R14 10.1186/gb-2010-11-2-r14 PMC2872874 20132535

[37] Livak KJ, Schmittgen TD. Analysis of relative gene expression data using real-time quantitative PCR and the 2^-ΔΔCT^ method. Methods. 2011:25(4): 402–408. 10.1006/meth.2001.1262 11846609

[38] Leitner A, Jensen-Jarolim E, Grimm R, Wuthrich B, Ebner H, Scheiner O, et al Allergens in pepper and paprika immunologic investigation of the celery-birch-mugwort-spice syndrome. Allergy. 1998;53: 36–41 10.1111/j.1398-9995.1998.tb03771.x 9491227

[39] Burk AW, Brooks JR, Sarnpson HA. J. Allergenicity of major component proteins of soybean determined by enzyme-linked immunosorbent assay (ELISA) and immunoblotting in children with atopic dermatitis and positive soy challenges. Allergy Clin Immunol. 1988;81(6): 1135–1142 10.1016/0091-6749(88)90881-0 3379225

[40] Rabjohn P, Helm EM, Stanley JS, West CM, Sampson HA, Burks AW, et al Molecular cloning and epitope analysis of the peanut allergen Ara h 3. Clin Invest. 1999;103 (4): 535–542 10.1172/JCI5349 PMC408104 10021462

[41] Davies CS, Coates JB, Nielsen NC. Inheritance and biochemical analysis of four electrophoretic variants of β-conglycinin from soybean.Theor Appl Genet. 1985;71: 351–358. 10.1007/BF00252079 24247406

[42] Scallon B, Thanh VH, Floener LA, Nielsen NC. Identification and characterization of DNA clones encoding group-II glycinin subunits. Theor Appl Genet. 1985;70: 510–519. 10.1007/BF00305984 24253061

[43] Nielsen NC, Dickinson CD, Cho TJ, Thanh VH, Scallon BJ, Fischer RL, et al Characterization of the glycinin gene family in soybean. Plant Cell. 1989;1: 313–328. 2485233 10.1105/tpc.1.3.313 PMC159764

[44] Beilinson V, Chen Z, Shoemaker RC, Fischer RL, Goldberg RB, Nielsen NC. Genomic organization of glycinin genes in soybean. Theor Appl Genet. 2002;104: 1132–1140. 12582623 10.1007/s00122-002-0884-6

[45] Muehlbauer GJ, Specht JE, Thomas-Compton MA, Staswick PE, Bernard RL. Near-isogenic lines a potential resource in the integration of conventional and molecular marker linkage maps. Crop Sci. 1988;28: 279–735.

[46] Dunwell JM. Cupins: a new superfamily of functionally diverse proteins that include germins and plant storage proteins. Biotechnol Genet Eng Rev. 1998;15: 1–32. 9573603 10.1080/02648725.1998.10647950

[47] Dunwell JM. Microbial relatives of seed storage proteins: conservation of motifs in a functionally diverse superfamily of enzymes. J Mol Ebol. 1998;46: 147–154. 10.1007/pl00006289 9452516

[48] Dunwell JM, Culham A, Carter CE, Sosa-Aguirre CR, Goodenough PW. Evolution of functional diversity in the cupin superfamily. Trends Biochem Sci. 2001;26: 740–746. 11738598 10.1016/s0968-0004(01)01981-8

[49] Dunwell JM, Purvis A, Khuri S. Cupins: the most functionally diverse protein superfamily? Phytochemistry. 2004;65: 7–17. 14697267 10.1016/j.phytochem.2003.08.016

[50] Meinke DW, Chen J, Beachy RN. Expression of storage-protein genes during soybean seed development. Planta. 1981;153: 130–139. 10.1007/BF00384094 24276763

[51] Niselsen NC, Bassuner R, Beaman TW. The biochemistry and cell biology of embryo storage proteins In: Larkins BA, Vasil IK, editors. Cellular and molecular biology of plant seed development. Dordrecht: Kluwer Academic Publishers; 1997 pp. 151–220.

